# Long-term mortality of acetaminophen poisoning: a nationwide population-based cohort study with 10-year follow-up in Taiwan

**DOI:** 10.1186/s13049-017-0468-8

**Published:** 2018-01-08

**Authors:** Hung-Sheng Huang, Chung-Han Ho, Shih-Feng Weng, Chien-Chin Hsu, Jhi-Joung Wang, Shih-Bin Su, Hung-Jung Lin, Chien-Cheng Huang

**Affiliations:** 1 Department of Emergency Medicine, Chi-Mei Medical Center, 901 Zhonghua Road, Yongkang District, Tainan City, 710 Taiwan; 2 Department of Occupational Medicine, Chi-Mei Medical Center, Tainan, Taiwan; 3 Department of Medical Research, Chi Mei Medical Center, Tainan, Taiwan; 4 Department of Hospital and Health Care Administration, Chia Nan University of Pharmacy and Science, Tainan, Taiwan; 5 Department of Healthcare Administration and Medical Informatics, Kaohsiung Medical University, Kaohsiung, Taiwan; 6 Department of Biotechnology, Southern Taiwan University of Science and Technology, Tainan, Taiwan; 7 Department of Leisure, Recreation, and Tourism Management, Southern Taiwan University of Science and Technology, Tainan, Taiwan; 8 Department of Medical Research, Chi Mei Medical Center, Liouying, Tainan, Taiwan; 9 Department of Emergency Medicine, Taipei Medical University, Taipei, Taiwan; 10 Department of Environmental and Occupational Health, College of Medicine, National Cheng Kung University, Tainan, Taiwan; 11 Bachelor Program of Senior Service, Southern Taiwan University of Science and Technology, Tainan, Taiwan; 12 Department of Geriatrics and Gerontology, Chi-Mei Medical Center, Tainan, Taiwan

**Keywords:** Acetaminophen, Long-term, Mortality, Poisoning

## Abstract

**Background:**

The long-term mortality of acetaminophen (APAP) poisoning has not yet been well studied; hence, we conducted this study to gain understanding of this issue.

**Methods:**

We conducted a nationwide population-based cohort study by identifying 3235 participants with APAP poisoning and 9705 participants without APAP poisoning in Taiwan between 2003 and 2012 in the Nationwide Poisoning Database and Longitudinal Health Insurance Database 2000. Participants with APAP poisoning and control subjects were compared for the risk of all-cause mortality by follow-up until 2013.

**Results:**

Two hundred forty-one participants with APAP poisoning (7.5%) and ninety-four control subjects (1.0%) died during the follow-up. Participants with APAP poisoning had a higher risk of all-cause mortality than the control subjects (incidence rate ratio [IRR], 8.1; 95% confidence interval [CI], 6.3–10.2), especially in the subgroup aged 20 years and younger (IRR, 27.3; 95% CI, 3.5–215.5) and in the first 12 months after poisoning (IRR, 16.0; 95% CI, 9.9–25.7). The increased risk of all-cause mortality was found even up to 2 years after the index poisoning.

**Conclusion:**

APAP poisoning was associated with increased long-term mortality. Early referral for intensive aftercare and associated interventions are suggested; however, further studies of the method are needed for clarification.

## Background

Acetaminophen (APAP) is one of the most commonly used antipyretics and analgesics. It can be bought in either pharmacy or nonpharmacy outlets or prescribed by physicians. Like all drugs, APAP is effective and safe when used at recommended doses, less than a daily maximum of dose 4 g/day in an adult; however, its overdose can result in toxicity [1]. Some of the negative outcomes of APAP overdose are hepatotoxicity, acute liver failure, and even death [2]. In spite of available antidote treatment with *N*-acetylcysteine, the morbidity and mortality of APAP poisoning are still high. According to a recent study in the United States, APAP poisoning is one of the leading causes of acute liver failure and results in over 30,000 hospitalizations annually [3]. The American Association of Poison Control Centers National Poison Data System in 2012 reported that APAP alone and APAP combination products were the fourth and sixth most common causes of substance poisoning-related casualties, respectively [4].

There is not much data on long-term mortality of APAP poisoning, because previous studies were focused mainly on short-term mortality, risk factors of complications, and management of APAP poisoning [5–7]. In a population-based study about the risk factors of complications, unintentional overdoses, alcohol abuse, and underlying liver disease were independently associated with hepatotoxicity [6]. In addition, previous studies were conducted on the basis of an assumption that the liver, the major organ affected by APAP poisoning, is capable of complete regeneration, leading to clinical recovery [8, 9]. There are only a few studies in the literature about long-term mortality associated with APAP poisoning. In a study done in a Danish national referral center during 1984–2004, researchers reported that the long-term mortality in APAP-induced acute liver failure (international normalized ratio > 1.7) was not different from APAP poisoning without acute liver failure [10]. However, in another study in Canada between 1995 and 2004, researchers reported the opposite result. That study showed that in APAP poisoning, the patients with acute hepatotoxicity (hepatic necrosis, toxic hepatitis, or hepatic encephalopathy) had a nearly threefold greater risk of mortality than those without acute hepatotoxicity during follow-up [6]. In addition to the inconsistent findings described above, both studies were focused on the comparison of mortality between patients with and without hepatotoxicity. The comparative mortality risk between patients with and without APAP poisoning has not been studied, and therefore we conducted this nationwide population-based cohort study with the objective of investigating the long-term mortality risk associated with APAP poisoning.

## Methods

### Data sources

The Nationwide Poisoning Database (NPD) and Longitudinal Health Insurance Database 2000 (LHID2000) were used for this study. These are the two data subsets of the National Health Insurance Research Database (NHIRD). The Taiwan National Health Insurance program covers 99% of whole population (23.3 million) in Taiwan, and its dataset, NHIRD, is one of the most complete, biggest, and most comprehensive databases in the world [11]. The NHIRD contains all information of the insured people, including basic sociodemographic characteristics; monthly income; dates of visit and discharge; diagnosis by International Classification of Diseases, Ninth Revision, Clinical Modification (ICD-9-CM), codes; treatments; and prescribed medications. The NPD contains all poisoning data, including APAP poisoning between 1999 and 2013. The LHID2000 contains all claims data of 1 million beneficiaries (4.34% of the total population) who were selected randomly from the NHIRD in 2000 [12]. There was no significant difference in gender distribution between NHIRD and LHID2000 after validation [12]. All medical expenditures related to APAP poisoning were paid by National Health Insurance.

### Participants and study design

All the participants who had been diagnosed with APAP poisoning (ICD-9 code 965.4 or E850.4) in the NPD between January 1, 2003, and December 31, 2012, were identified as the study participants in the APAP group. In addition to APAP poisoning, participants in the APAP group may have had other diagnoses at the same time. The diagnosis of APAP poisoning depends on the history of taking APAP, blood APAP level, and the treating physician’s high index of suspicion [13, 14]. In the control group, participants were individuals without APAP poisoning randomly identified from the LHID2000 by matching age, sex, and index date with the APAP group in a 3:1 ratio. The index date was defined as the date when a participant was diagnosed as having APAP poisoning. Participants in the APAP group were excluded if they were diagnosed with APAP poisoning before January 1, 2003. Variables including age, sex, comorbidities, geographic region, and monthly income were included for the analysis. Comorbidities were defined as follows: diabetes mellitus (DM; ICD-9 codes 250.0–250.93), coronary artery disease (CAD; ICD-9 codes 410–414), stroke (ICD-9 codes 433–436), hypertension (HTN; ICD-9 codes 401–405), liver disease (ICD-9 codes 570–576), renal disease (ICD-9 codes 580–593), mental disorder (ICD-9 codes 290–302, 306–319), and cancer (ICD-9 codes 140–208). The comorbidities were included in the analysis if the participant had the comorbidity at admission or during outpatient care at least two times before the index date. Both groups were followed from the index date until death or December 31, 2013, the end date of the study. According to Taiwanese law, all citizens or people owning a residence permit must participate in the National Health Insurance program, and they must be removed from the National Health Insurance program within 30 days after death. Therefore, we defined death in cases where the patient had been registered as dead (ICD-9 code 798) or had been withdrawn from the National Health Insurance program. Because the insurance is compulsory with a long grace period for premium payment, almost all participants who were withdrawn from the NHIRD represented unreported deaths.

### Ethics statement

This study was approved by the institutional review board at Chi-Mei Medical Center and conducted according to the principles of the Declaration of Helsinki. Because the two datasets used in this study contain unidentified information of the participants, the need for informed consent was waived.

### Statistical analysis

A two-sample *t* test and the chi-square test were used for the comparison of continuous variables and categorical variables between participants with APAP poisoning and control subjects, respectively. We used Poisson regression to calculate the incidence rate ratio (IRR) of the risk of all-cause mortality between the two groups. Kaplan–Meier analysis and the log-rank test were used for comparison of cumulative survival rates between the two groups. We also investigated the independent mortality predictors by Cox proportional hazards regression. SAS 9.4 for Windows software (SAS Institute, Cary, NC, USA) was used for all the analyses. The significance level was set at *p* < 0.05 (two-tailed).

## Results

A total of 3235 participants with APAP poisoning and 9705 control subjects were identified for inclusion in the study (Table 1). The mean (± standard deviation) age of the APAP group and control group was 31.3 (±13.4) years (Table 1). In both groups, the 21- to 39-year-old age group comprised 69.1% of the sample, and 72.3% of participants were female. Participants with APAP poisoning had a higher prevalence of CAD, liver disease, renal disease, mental disorders, and cancer than the control subjects (Table 1). Most participants in both groups lived in the northern area of Taiwan (93.1% versus 91.5%, respectively). Participants with APAP poisoning had a lower monthly income than the control subjects.

**Table 1:** Comparison of demographic data and comorbidities between APAP group and control subjects

Variable	APAP group (*n* = 3235)	Control group (*n* = 9705)	*p* Value
Age at index date (years)	31.3 ± 13.4	31.3 ± 13.4	>0.999
Age at index date (years)			
0–20	385 (11.9)	1155 (11.9)	>0.999
21–39	2235 (69.1)	6705 (69.1)	
40–64	489 (15.1)	1467 (15.1)	
≥ 65	126 (3.9)	378 (3.9)	
Sex			
Male	895 (27.7)	2685 (27.7)	>0.999
Female	2340 (72.3)	7020 (72.3)	
Comorbidity^a^			
DM	124 (3.8)	313 (3.2)	0.097
CAD	149 (4.6)	317 (3.3)	<0.001
Stroke	84 (2.6)	137 (1.4)	<0.001
HTN	248 (7.7)	666 (6.9)	0.122
Liver disease	396 (12.2)	952 (9.8)	<0.001
Renal disease	308 (9.5)	633 (6.5)	<0.001
Mental Disorder	1139 (35.2)	1409 (14.5)	<0.001
Cancer	75 (2.3)	145 (1.5)	0.002
Geographic region			
North	3013 (93.1)	8876 (91.5)	0.019
Center	92 (2.8)	315 (3.3)	
South	121 (3.7)	479 (4.9)	
East	9 (0.3)	35 (0.4)	
Monthly income			
NTD ≤ 15,840	1655 (51.2)	3509 (36.2)	<0.001
NTD 15841–25,000	1152 (35.6)	3975 (41.0)	
NTD ≥ 25,001	428 (13.2)	2221 (22.9)	

Data are presented as n (%) or mean ± standard deviation. *APAP* Acetaminophen, *DM* Diabetes mellitus, *CAD* Coronary artery disease, *HTN* Hypertension, *NTD* New Taiwan dollar. ^a^Participant may have multiple comorbidities

**Fig. 1:**
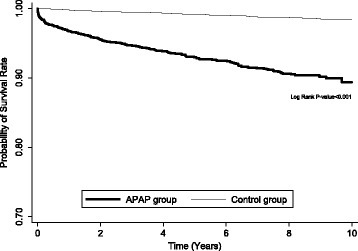
Kaplan–Meier survival curve and log rank test for participants with APAP poisoning and control subjects during the follow-up. APAP, acetaminophen

During the follow-up period, the all-cause mortality of the participants with APAP poisoning and control subjects was 7.5% (241 of 3235) and 1.0% (94 of 9705), respectively (Table 2). The participants with APAP poisoning had higher risk of all-cause mortality than control subjects (IRR, 8.1; 95% confidence interval [CI], 6.3–10.2) (Table 2). In the subgroup aged 20 years or younger, participants with APAP poisoning had a higher risk of all-cause mortality than control subjects (IRR, 27.3; 95% CI, 3.5–215.5), followed by those aged 21–39 years, 40–64 years, and 65 years or older. In both sexes, participants with APAP poisoning had a higher risk of all-cause mortality than the control subjects (male, IRR, 9.2; 95% CI, 6.5–13.0; female, IRR, 7.3; 95% CI, 5.3–10.1). In all stratified analyses by baseline comorbidities, participants with APAP poisoning had a higher risk of all-cause mortality than the control subjects. During the whole follow-up period, participants with APAP poisoning had a higher risk of all-cause mortality than the control subjects, especially in the first 12 months after the index APAP poisoning (IRR, 16.0; 95% CI, 9.9–25.7). Kaplan–Meier survival analyses and log-rank tests also showed higher risk of all-cause mortality in the participants with APAP poisoning than in control subjects during the follow-up period (Fig. 1).

**Table 2:** Comparison of the risk of morality between APAP group and control group

Variable	APAP group				Control group				IRR (95% CI)	*p*-value
	n	Death	PY#	Rate*	n	Death	PY#	Rate*		
All	3235	241	17,685.4	13.6	9705	94	55,497.5	1.7	8.1 (6.3–10.2)	<0.001
Age (years)										
≤20	385	9	2276.7	4.0	1155	1	6905.8	0.1	27.3 (3.5–215.5)	0.002
21–39	2235	119	12,768.9	9.3	6705	23	39,663.3	0.6	16.1 (10.3–25.1)	<0.001
40–64	489	58	2259.2	25.7	1467	27	7358.4	3.7	7.0 (4.4–11.1)	<0.001
≥ 65	126	55	380.5	144.5	378	43	15,670.0	27.4	5.3 (3.5–7.9)	<0.001
Sex										
Male	895	121	4601.9	26.3	2685	43	14,969.4	2.9	9.2 (6.5–13.0)	<0.001
Female	2340	120	13,083.6	9.2	7020	51	40,528.1	1.3	7.3 (5.3–10.1)	<0.001
Comorbidity†										
DM	124	37	397.6	93.1	313	23	1331.5	17.3	5.4 (3.2–9.1)	<0.001
CAD	149	48	540.2	88.9	317	26	1346.4	19.3	4.6 (2.9–7.4)	<0.001
Stroke	84	27	323.6	83.4	137	22	501.6	43.9	1.9 (1.1–3.3)	0.025
HTN	248	71	912.0	77.9	666	47	2918.3	16.1	4.8 (3.3–7.0)	<0.001
Liver disease	396	54	1691.6	31.9	952	38	4633.6	8.2	3.9 (2.6–5.9)	<0.001
Renal disease	308	46	1338.5	34.4	633	18	3099.2	5.8	5.9 (3.4–10.2)	<0.001
Mental Disorder	1139	106	5379.8	19.7	1409	42	6831.1	6.2	3.2 (2.2–4.6)	<0.001
Cancer	75	28	249.1	112.4	145	13	683.1	19.0	5.9 (3.1–11.4)	<0.001
Follow up period										
0–12 months	3235	104	3042.2	34.2	9705	20	9329.4	2.1	16.0 (9.9–25.7)	<0.001
1–2 year	2897	34	2761.6	12.3	8958	19	8576.6	2.2	5.6 (3.2–9.7)	<0.001
≥ 2 year	2641	103	11,881.6	8.7	8236	55	37,591.5	1.5	5.9 (4.3–8.2)	<0.001

#PY, person-years. *Rate, per 1000 person-years. †Participant may have multiple comorbidities. APAP, acetaminophen; IRR, incidence rate ratio; CI, confidence interval; DM, diabetes mellitus; CAD, coronary artery disease; HTN, hypertension

The results of Cox proportional hazards regression analysis showed that in addition to APAP poisoning, older age, male sex, DM, liver disease, cancer, living in the eastern geographic region of Taiwan, and lower monthly income were associated with higher risk of all-cause mortality after adjusting for all the variables (Table 3).

**Table 3:** Independent mortality predictors in all participants by Cox proportional hazards regression

Variable	Crude Hazard Ratio (95% CI)	Adjusted Hazard Ratio (95% CI)^a^
APAP poisoning		
Yes	8.0 (6.3–10.2)	7.4 (5.8–9.6)
No	1.0	1.0
Age (years)		
≤ 20	1.0	1.0
21–39	2.5 (1.3–4.7)	3.0 (1.5–5.6)
40–64	7.8 (4.1–15.1)	6.2 (3.2–12.3)
≥ 65	41.4 (21.6–79.5)	14.9 (7.2–30.9)
Sex		
Male	2.6 (2.1–3.2)	2.1 (1.6–2.6)
Female	1.0	1.0
DM		
Yes vs. No	8.3 (6.2–10.9)	1.6 (1.2–2.3)
CAD		
Yes vs. No	1.0 (7.7–12.9)	1.3 (0.9–1.8)
Stroke		
Yes vs. No	13.5 (1.0–18.3)	1.2 (0.8–1.7)
HTN		
Yes vs. No	9.1 (7.3–11.4)	1.5 (1.0–2.1)
Liver disease		
Yes vs. No	3.8 (3.0–4.8)	1.4 (1.1–1.9)
Renal disease		
Yes vs. No	3.5 (2.7–4.6)	1.1 (0.8–1.4)
Mental Disorder		
Yes vs. No	3.8 (3.0–4.7)	1.28 (0.9–1.5)
Cancer		
Yes vs. No	10.1 (7.3–14.0)	2.6 (1.8–3.6)
Geographic region		
Northern	0.2 (0.1–0.8)	0.1 (0.03–0.3)
Central	0.2 (0.04–0.8)	0.1 (0.03–0.5)
Southern	0.3 (0.1–1.1)	0.2 (0.04–0.5)
Eastern	1.0	1.0
Monthly income		
NTD ≤ 15,840	4.5 (2.9–6.9)	2.2 (1.4–3.5)
NTD 15840–25,000	2.3 (1.5–3.7)	1.6 (1.0–2.5)
NTD ≥ 25,001	1.0	1.0

^a^Adjusted for all the variables. *APAP* Acetaminophen, *CI* Confidence interval, *DM* Diabetes mellitus, *CAD* Coronary artery disease, *HTN* Hypertension, *NTD* New Taiwan dollar

## Discussion

This was the first nationwide population-based cohort study delineating long-term risk of all-cause mortality associated with APAP poisoning. In comparison with control subjects, participants with APAP poisoning had a higher risk of all-cause mortality, especially in the younger population and in the first 12 months after poisoning. The increased risk of all-cause mortality in the participants with APAP poisoning compared with control subjects was observed even 2 years after the poisoning. In addition to APAP poisoning, older age, male sex, DM, liver disease, cancer, living in the eastern geographic region of Taiwan, and lower monthly income were also independent mortality predictors.

A possible explanation for the increased long-term all-cause mortality in participants with APAP poisoning is that the related oxidative stress causes damage to the pancreas, liver, kidney, vascular walls, and other organs [15]. The APAP-induced hepatocellular injury is a dose-related response [15] with *N*-acetyl-*p*-benzoquinone imine (NAPQI), the reactive metabolite of APAP, as the main source of toxicity [16]. Intake of high amounts of APAP leads to production of high NAPQI along with hepatic and renal toxic metabolites, resulting in subendothelial damage, oxidative stress, and an insufficient amount of glutathione, which contribute to hepatic and renal toxicity and even death [17, 18].

This study shows that the risk of all-cause mortality after APAP poisoning was higher in the younger population than in the older population. A possible explanation is that the younger population has fewer comorbidities than the older population, and therefore APAP poisoning may become the major contributor to death. This finding is consistent with results of previous studies on poisoning [19, 20]. The increased all-cause mortality was higher during the whole follow-up period and was highest in the first 12 months after APAP poisoning. This indicates that APAP poisoning was associated more with short-term mortality than with long-term mortality. The 95% CI of the IRR in the subgroup aged 20 years and younger was very wide (3.5–215.5), which suggests poor precision of measurement. Therefore, the results need to be interpreted carefully.

Older age and male sex were independent mortality predictors, which is consistent with previous studies [21–23]. In a nationwide study in Taiwan in 2011, researchers reported that males aged 65 years or older were at highest risk of poisoning and that male participants had a higher mortality rate than female participants (57.6% versus 26.2%) [21]. In a hospital-based study of 1512 poisoning cases in Taiwan, researchers reported that patients aged 61 years or older had a mortality rate 4.3 times higher than those younger than 61 years of age [22]. Another nationwide study in Taiwan that included unintentional poisoning cases also revealed that patients aged over 65 years had a 14.83 times higher inpatient mortality than children aged between 0 and 14 years [23]. Elderly individuals have poor physiological condition, more comorbidities, and altered toxicokinetics and toxicodynamics, which may contribute to poorer outcomes than in the younger population [23, 24].

The present study shows that participants with APAP poisoning had a lower monthly income and a higher prevalence of mental disorders than those without APAP poisoning, which is compatible with findings of a previous study [25]. In a study of 1543 patients in Canada between 1995 and 2004, researchers reported that low socioeconomic status was a risk factor for APAP poisoning [25]. There was also a fourfold higher risk of hospitalization among patients receiving social assistance than among those without it [25]. Fifty-five percent patients had a history of depression, and 85% APAP poisonings were intentional [25]; therefore, it is likely that the risk factors themselves are actually more important than poisoning and that poisoning is just a symptom of risky behavior. In another study by the World Health Organization, researchers also reported that lower income was associated with increased mortality resulting from poisoning [26]. The probable reason explained in that report was that people with lower income may have limited access to medical resources and a lower standard of living, which may contribute to higher risk of death after poisoning [26]. Despite the fact that National Health Insurance covers nearly all of the Taiwanese population, people with lower income may live more remote from medical resources than those with higher income. This also explains the finding in our study that participants living in the eastern part of Taiwan had higher all-cause mortality than those living in other areas.

In this study, a direct comparison of all-cause mortality between participants with and without APAP poisoning was performed, which was different from two previous studies in which researchers compared mortality among subgroups of APAP poisoning [6, 10]. A study done in a Danish national referral center during 1984–2004 included 641 patients, and the researchers examined whether APAP-induced acute liver failure increased long-term mortality [10]. They found that APAP-induced acute liver failure did not affect long-term mortality [10]. In another study done in Canada between 1995 and 2004, researchers included 1543 patients and examined outcomes of APAP overdose [6]. They found that 1% of patients died in the hospital and that the risk factors were older age; unintentional overdoses; alcohol abuse; comorbidities, including liver disease; and hepatotoxicity [6]. During a median follow-up of 5.2 years, 5.1% of patients died [6]. The most common causes of death were suicide, substance abuse, and trauma [6]. The patients with acute hepatotoxicity had a nearly threefold greater risk of mortality than those without acute hepatotoxicity during follow-up [6].

This nationwide cohort study has two major strengths: (1) a large sample size and (2) insights into an area with limited data. However, there are some limitations as well. First, some information, including body mass index, lifestyle factors, smoking, substance abuse, physical activity, family history, suicide attempts, causes of poisoning (i.e., intended versus unintended poisoning), concomitant poisoning, and causes of mortality, was not included in this study, which might affect the causal relationship between APAP poisoning and mortality. For example, concomitant poisoning may be more dangerous than APAP poisoning, and the increased mortality in the young patients may be due to accidents or suicide. We adjusted for many major comorbidities that can be surrogates for some unavailable variables above. For example, DM and HTN were adjusted for body mass index and lifestyle factors, and liver disease was adjusted for substance abuse. Therefore, we believe the influence of missing comorbidities to be minimal. Second, this study provides only diagnosis codes of APAP poisoning, regardless of severity, including development into liver injury and hepatotoxicity or not. Third, we did not evaluate the association between *N*-acetylcysteine and mortality in this study. Fourth, although this was a nationwide study, it may not be generalizable to other nations, owing to differences in race, medical resources, and culture. All the limitations mentioned above warrant further studies to provide more insight into this area.

## Conclusions

APAP poisoning was associated with increased long-term all-cause mortality. The increased all-cause mortality was more prominent in the younger population and in the first 12 months after poisoning. Independent mortality predictors included older age, male sex, DM, liver disease, cancer, lower monthly income, and living in the eastern region of Taiwan. Early recognition of APAP poisoning and timely management of the above-mentioned risk factors are suggested to prevent subsequent death. Further studies investigating the severity of APAP poisoning, causes of poisoning, use of *N*-acetylcysteine, causes of mortality, and methods of aftercare are warranted.
